# Is Beta Radiation Better than 5 Flurouracil as an Adjunct for Trabeculectomy Surgery When Combined with Cataract Surgery? A Randomised Controlled Trial

**DOI:** 10.1371/journal.pone.0161674

**Published:** 2016-09-08

**Authors:** Kazim Dhalla, Simon Cousens, Richard Bowman, Mark Wood, Ian Murdoch

**Affiliations:** 1 Department of Ophthalmology, Comprehensive Community Based Rehabilitation Hospital in Tanzania (CCBRT), Dar Es Salaam, Tanzania; 2 Department of Infectious Disease Epidemiology, London School of Hygiene and Tropical Medicine (LSHTM), London, United Kingdom; 3 International Centre for Eye Health, London School of Hygiene and Tropical Medicine (LSHTM), London, United Kingdom; 4 Institute of Ophthalmology, University College of London, London, United Kingdom; Universita degli Studi di Firenze, ITALY

## Abstract

**Introduction:**

In an African setting surgery is generally accepted as the treatment of first choice for glaucoma. A problem with trabeculectomy surgery for the glaucomas is the frequent co-existence and exacerbation of cataract. We report a randomized controlled trial to compare the use of beta radiation with 5FU in combined cataract and glaucoma surgery.

**Participants and Methods:**

Consenting adults aged >40 years with glaucoma, an IOP>21mmHG and cataract were enrolled and randomised to receive either 1000cG β radiation application or sub-conjunctival 5fluorouracil (5FU) at the time of combined trabeculectomy and phaco-emulsification with lens implant surgery.

**Results:**

385 individuals were eligible for inclusion of whom 301 consented to inclusion in the study (one eye per patient). 150 were randomised to the 5FU arm and 151 received β radiation. In the 12 months following surgery there were 40 failures (IOP>21mmHg) in the 5FU arm and 34 failures in the beta arm. The hazard ratio for the beta radiation arm compared to the 5FU arm, adjusted for IOP at baseline, was 0.83 (95% c.i. 0.54 to 1.28; P = 0.40). The improvement from mean presenting visual acuities of 0.91 and 0.86 logMAR to 0.62 and 0.54 in the 5FU and beta arms respectively was comparable between groups (P = 0.4 adjusting for baseline VA). Incidence of complications did not differ between the two groups.

**Discussion:**

This study highlights several important issues in the quest for a therapeutic strategy for the glaucomas in an African context. Firstly, there is no evidence of an important difference between the use of 5FU and beta radiation as an anti-metabolite in phacotrabeculectomy. Secondly phacotrabeculectomy is a successful operation that improves visual acuity as well as controlling IOP in a majority of patients. Although the success of trabeculectomy in lowering IOP is reduced when combined with phacoemulsification compared with trabeculectomy alone, this finding has to be set against the possible need for subsequent cataract surgery following trabeculectomy alone, which represents a second trip and expense for the patient and results in 10–61% failure of the trabeculectomy at one year post-cataract surgery.

**Trial Registration:**

ISRCTN Registry ISRCTN36436933

## Introduction

In 2010 285 million people in the world were estimated to be visually impaired, with 39 million of those blind [1]. More than 80% of these visually impaired individuals live in low and middle income countries, with 15% in Africa. This continent has only 12% of the world’s population and the population is relatively young. Two recent large population based surveys in Nigeria and Kenya identified the glaucomas as the second most common cause of blindness after cataract [2,3].

Like cataract there are treatments for the glaucomas, but unlike cataract there is not an operation that returns sight to the blind. Glaucomas have been called the ‘thief of sight’ because the disease is not noticed until major visual loss has occurred. Loss of sight is irreversible and thus treatment is preventive not curative.

The principal treatment approach for the glaucomas is to lower the pressure in the eye with a view to preventing further optic nerve damage. This can be achieved by use of regular topical or systemic medication, laser treatment or surgery. In an African setting surgery is generally accepted as the treatment of first choice[4]. The most common surgical procedure is trabeculectomy surgery. However, in African patients a successful outcome of glaucoma surgery is often compromised by an aggressive healing response[5,6].For this reason anti-metabolites may be used, most commonly 5 fluorouracil (5FU) or mitomycin C (MMC)[7]

Beta-radiation has features that make it suitable for use in glaucoma surgery. Application is rapid and simple: both the dose and the area treated can be controlled with accuracy. Furthermore, the beta-radiation delivery device, once purchased, is self-sterilising and has a long working life (20 years+) with minimal maintenance required. In settings where a regular supply of drugs and maintenance of equipment are not assured, this makes beta-radiation particularly attractive. Four randomised controlled trials of β-radiation in trabeculectomy have been published. These show that use of beta-radiation with trabeculectomy surgery results in a lower risk of surgical failure compared to trabeculectomy surgery alone. A Cochrane review concluded that a trial of beta radiation versus anti-metabolites is warranted [8].

At present, prevention of blindness from the glaucomas faces several challenges in low and middle income settings. A major problem lies in getting those who present to eye departments with the disease to accept treatment. Compliance with glaucoma surgery at our hospital (Comprehensive Community Based Rehabilitation and Training Hospital (CCBRT) in Dar-Es-Salaam, Tanzania) is only 48% [9]. Patients usually arrive blind in one eye and with severe disease in the other. They are extremely concerned when offered no hope for the blind eye and surgery to their ‘good eye’. Furthermore the surgery does not improve vision but rather may make vision temporarily worse in the longer term interest of preserving that vision. This outcome of ‘vision saved’ rather than ‘vision restored’ is extremely difficult to present to and get accepted by patients.

An additional problem with trabeculectomy surgery for the glaucomas is the frequent co-existence and exacerbation of cataract[10]. In an African context, if the cataract does not cause significant disability it would not normally warrant surgical intervention. Removal of the cataract would, none-the-less, offer brighter vision and avoid the need for surgery at a later date. Offering patients improved vision might make patients more willing to accept glaucoma surgery.

Despite extensive discussion of combined cataract and trabeculectomy surgery in the literature, there are remarkably few papers comparing trabeculectomy surgery and phacoemulsification combined with trabeculectomy surgery alone (Table 1), and none are from the African continent. One study in Japan reported considerably worse outcomes after combined surgery. The other studies do not suggest a major difference between the two procedures. All studies reported a deterioration in visual acuity 1 year after trabeculectomy alone. Unsurprisingly an improvement in visual acuity was seen for those having the combined procedure.[11–14]

**Table 1:** Studies comparing trabeculectomy surgery alone with combined phacoemulsification and trabeculectomy surgery.

Ref No.	Study	Design	N	F/Up	Results
11	• Long term effect on IOP of Phacotrabeculectomy versus trabeculectomy • UK 2003	• Retrospective Comparative	• 44 eyes	≥12 months	• Final mean IOP (mmHg) • Phacotrabeculectomy = 15.5 • Trabeculectomy = 13.0 (P = 0.0017) • Mean reduction in IOP • Phacotrabeculectomy = 6.7 • Trabeculectomy = 11 • Vision gain in Phacotrabeculectomy Vision loss in trabeculectomy
12	• Trabeculectomy with MMC and Phacotrabeculectomy with MMC • Japan 2013	• Prospective Comparative • Success criteria; reduction in IOP (mmHg) • A: ≤21 • B: ≤18 • C ≤ 15	• 52 eyes • 26:26 Phacotrabeculectomy/ trabeculectomy	1 year	• Post operative success • Trabeculectomy / Phacotrabeculectomy; • A: 96.2% / 72.9% • B: 96.2% / 61.1% • C: 80.4% / 46.2% • Visual improvement in Phacotrabeculectomy
13	• 5FU safety and efficacy in Trabeculectomy and Phacotrabeculectomy • Australia 2001	• Retrospective	• 186 eyes • 51/ 135 phacotrabeculectomy/ Trabeculectomy • Success IOP<16 mmHg and 30% reduction ± Medication	2 years	• Failure rate • Phacotrabeculectomy 39% • Trabeculectomy with 5FU 20% • Trabeculectomy without 5FU 46% • Visual improvement in Phacotrabeculectomy significant
14	• IOP outcome with Phacotrabeculectomy-5FU and Trabeculectomy-5FU • UK 2006	• Retrospective • Success • IOP<16 mmHg • IOP <20 ± medication	• 92 eyes • 45 phacotrabeculectomy/ • 47 trabeculectomy	2 years	• Mean IOP and(%)reduction: • Phacotrabeculectomy 16.1 (31.2) • Trabeculectomy 13.9 (44.6) • IOP <16 • Phacotrabeculectomy (62.2%) • Trabeculectomy (63.8%) • IOP <20 • Phacotrabeculectomy (93.3%) • Trabeculectomy (97.9%) • Visual acuity • Phacotrabeculectomy improved (62%) • Trabeculectomy worse (40%)

Current practice for combined surgery uses 5FU. However, this drug is known to cause corneal epithelial toxicity and endothelial compromise if it inadvertently enters the anterior chamber. In addition, use of this drug relies on a regular supply chain We undertook a retrospective review of the outcome of 163 combined cataract and trabeculectomy with 5FU operations in CCBRT Hospital. Of those seen at 6 months post operation, 16/19 (84% (95% c.i. 68–100%))[15] had an IOP of 6-20mmHG compared to 90% (95% c.i. 85–95%) in a previous audit of trabeculectomy surgery alone[16]. 85% (95% c.i. 64–95%) had improved visual acuity following the combined procedure

In order to further investigate the optimal management strategy for glaucoma therapy in an African context we undertook a randomized controlled trial to compare the use of beta radiation with 5FU in combined cataract and glaucoma surgery.

## Participants and Methods

We performed a randomised controlled trial of combined phacoemulsification and trabeculectomy surgery using 1000cG β radiation application in one arm and 5 Fluorouracil in the other at the CCBRT hospital in Tanzania. The study was, of necessity, open label in theatre. However, the outcome assessor was not aware of participants’ treatment assignment. Black African patients from the general ophthalmology clinic who met the referral criteria (Table 2) were given appointments for the Phacotrabeculectomy research clinic where the trial ophthalmologist (KD) examined them to determine if they met the trial’s inclusion criteria (Table 2), including assessing whether the cataract was sufficient to decrease vision and require surgical interventionand the patient was expected to benefit from the phacotrabeculectomy procedure. A cataract was considered to decrease vision and require surgical intervention if the affected eye had a best corrected visual acuity ≤ Snellen’s 6/12 (logMAR 0.3010) and a LOCS (Lens Opacities Grading System) III grade of ≥NC3, ≥ C4 or P3. Patients meeting the inclusion criteria had the purpose of the study and the procedures involved explained, and those who agreed to participate signed an informed consent form. Patients were frequently accompanied by a close relative who was also involved in the decision-making process.

**Table 2:** Referral, Inclusion and Exclusion criteria.

Referral criteria	Inclusion criteria	Exclusion criteria
• IOP >21mmHg • C/D ratio >0.8 • Age > 40 years • Any form of cataract	• Consent to inclusion and participation in trial. • Changes in the optic disc characteristic of glaucoma. The presence of a focal or diffuse area of optic disc rim loss, so that the neuroretinal rim tissue in any quadrant is less than 5% of the disc diameter in that meridian. Extensive loss of neuroretinal rim tissue with marked optic disc cupping giving a cup disc ratio greater than 0.8. • A measured intraocular pressure greater than 21 mmHg on at least one visit before the time of listing for surgery as measured by Goldmann applanation tonometry. • An open angle on gonioscopy • Cataract sufficient to decrease vision and require surgical intervention.	• Unwillingness to participate in the study • Anterior segment neovascularisation • Past trauma to the eye or ocular adnexae • Retinal or optic nerve neovascularisation • Aphakia or pseudophakia • Previous ocular surgery • Uveitis • Inability/unwillingness to give informed consent • Unwillingness to accept randomization • Pregnancy or female of childbearing age who may be pregnant at the time of treatment (LMP) • Long term use of topical or systemic steroids

All enrolled patients underwent a detailed clinical assessment. Visual acuity, pin hole acuity and best corrected visual acuity were measured using a reduced logarithm of minimum angle of resolution acuity chart (log Mar) by an optometrist who had been trained to use the chart. The tumbling E chart (log Mar) was used for unlettered patients. Intraocular pressure was measured using a Goldmann tonometer which was calibrated and checked at the beginning of each clinic. Three IOP readings were taken per patient at least 30 minutes apart. Patients who were on IOP lowering medication on referral were asked to stop the medication and to return after 2 weeks. Gonioscopy was done using a 4 mirror gonio lens and modified Shaffer gradings were recorded where a grade IV represents observation of the ciliary body; grade III indicates scleral spur; grade II indicates anterior trabecular meshwork; grade I Schwalbe’s line visible and grade 0 Schwalbe’s line not visible. Grade II and above was taken as open angle. Cataract was assessed after dilation using the LOCS III grading system[17]. Prior to the start of the trial, staff were trained by an external expert (JK) to minimize inter-observer variation for the key outcome variables of IOP and visual acuity. Training exercises were performed to standardize LOCS grading of the cataract and optic disc assessment. Visual field testing was not routinely performed. Phacotrabeculectomy surgical technique was left to the operating surgeon’s discretion.

### Randomization and masking

Patients were randomized to receive either β radiation or 5FU in blocks of 10 using computer generated random numbers. The randomised allocation was printed and sealed in an opaque envelope with the study number on the outside. The envelopes were kept in the theatre. The trial ophthalmologist (KD) was masked to the randomisation group since he neither operated on the patients nor attended the surgical sessions of the trial patients. The relevant sealed envelope (matching the patient’s study number) was opened after the phacoemulsification procedure by the surgeon’s assistant. At the completion of surgery the randomization slip was returned to the envelope and kept in the custody of the research assistant (IK). The randomization code was only known to IM, who was not involved in clinical care.

### Intervention procedures

For patients in the β radiation arm, the β probe disc was placed over the surgical site after conjunctival closure for sufficient time to administer 1000CGy. The radiation emitter contained strontium-90/yttrium-90 in an 8 mm disc applicator (SI-20; Nycomed Amersham, Buckinghamshire). Strontium -90 decays to yttrium-90 on release of the particle. A Tanzanian National Atomic Energy commission licence was obtained before using the probe and the licence renewed every year. For patients receiving 5FU, surgeons decided if they wanted to place 5FU soaked cotton pledgets under the bleb prior to trabeculectomy or give it as an injection subconjunctivally at the conclusion of surgery.

### Follow up

All patients were seen at the trial clinic post-operatively by the trial ophthalmologist (KD). On day 1 post-operatively they were all given a combination of dexamethasone and chloramphenicol eye drops for use four times a day for three months. Subsequent follow up examinations were scheduled for day 5–7, 1 month, 3 months, 6 months and 12 months post operatively. Log Mar visual acuities with and without pin hole correction were measured at all visits except day 1. Refraction was done at 3 and 12 months, IOP was measured at all visits and after the first day, IOP was measured twice with an interval between measurements of at least 30 minutes. A detailed postoperative assessment was done at each visit, which included bleb assessment, anterior chamber activity and morphology, status of the posterior capsule and fundoscopy. Any complications were noted and the operating surgeon was informed if any further surgical intervention was needed. At the 12 month follow-up, IOP was measured three times with at least a 30 minute interval between measurements and the bleb was assessed using the Moorfields Bleb Grading system (Refer to www.blebs.net website). An ongoing record was kept of subsequent reviews in the clinic.

### Outcomes

The primary outcome was surgical success at 12 months, defined as an intraocular pressure less than 16mmHg without ocular hypotensive treatment. To aid comparability with other studies in Africa, we also examined a definition of success as an IOP≤21 mmHG, with or without ocular hypotensive treatment. Secondary outcomes were time to IOP failure, visual acuity, and intra and postoperative complications.

### Statistical analysis

#### Sample size

To detect a clinically important increase in the success proportion from 75% to 90% with a power of 80% (5% significance level) we estimated that we required 113 patients per arm. Allowing for losses to follow-up of up to 25%, we estimated that we would need to enroll about 150 patients per arm (300 in total).

All data were recorded on trial proformas and double entered using EpiInfo v7.1.2.0 (http://wwwn.cdc.gov/epiinfo/7/). The two files were cross checked and any discrepancies resolved by reference to the original record. The data were then checked for consistency.

Analysis was undertaken using Stata version 14 (www.stata.com). The baseline characteristics of the patients in each arm were examined to check that the two arms were comparable. The proportions of patients with a successful outcome at each follow-up point were compared using the chi-squared test. Cumulative risk of failure was estimated using the Kaplan-Meier method, and the null hypothesis of no difference between the two arms was tested using the log rank test. Hazard ratios and 95% confidence intervals were estimated using Cox regression. Generalised estimating equations (GEE) with an identify link function was used to model the probability of success across all of the observations, accounting for within individual correlation. Visual acuity was analysed using linear regression. Proportions of patients experiencing complications were compared using the chi-squared test. Two-sided statistical tests were used throughout.

### Ethics

The study was approved by the Tanzanian research regulatory body, the National Institute for Medical Research (NIMR) and registered as NIMR/HQ/R.8a/Vol. IX/717. All patients gave free and informed written consent (signature or thumb print) prior to enrolment in the study. Patient recruitment took place between 9^th^ April 2009 and 18^th^ October 2012. The trial was registered with the ISRCT on 30^th^ November 2009, ISRCTN36436933. The delay in commencing patient recruitment from the time ethical clearance was granted (4^th^ August 2008) was because of logistical challenges which included importation of radioactive material in to Tanzania, inspection of the radioactive applicator by the Radiation control office, training of staff handling and storing the applicator and putting the trial systems in place. A preoccupation with these trial logistics also led to delay in registering the trial with the ISRCT. The trial stopped enrolling patients once the sample size was achieved and the trial was closed once the last patient enrolled completed the one year follow up assessment. The full trial protocol is available on request from the Phacotrabeculectomy β-project research team through the corresponding author. There are no ongoing trials at present.

## Results

**Fig 1:**
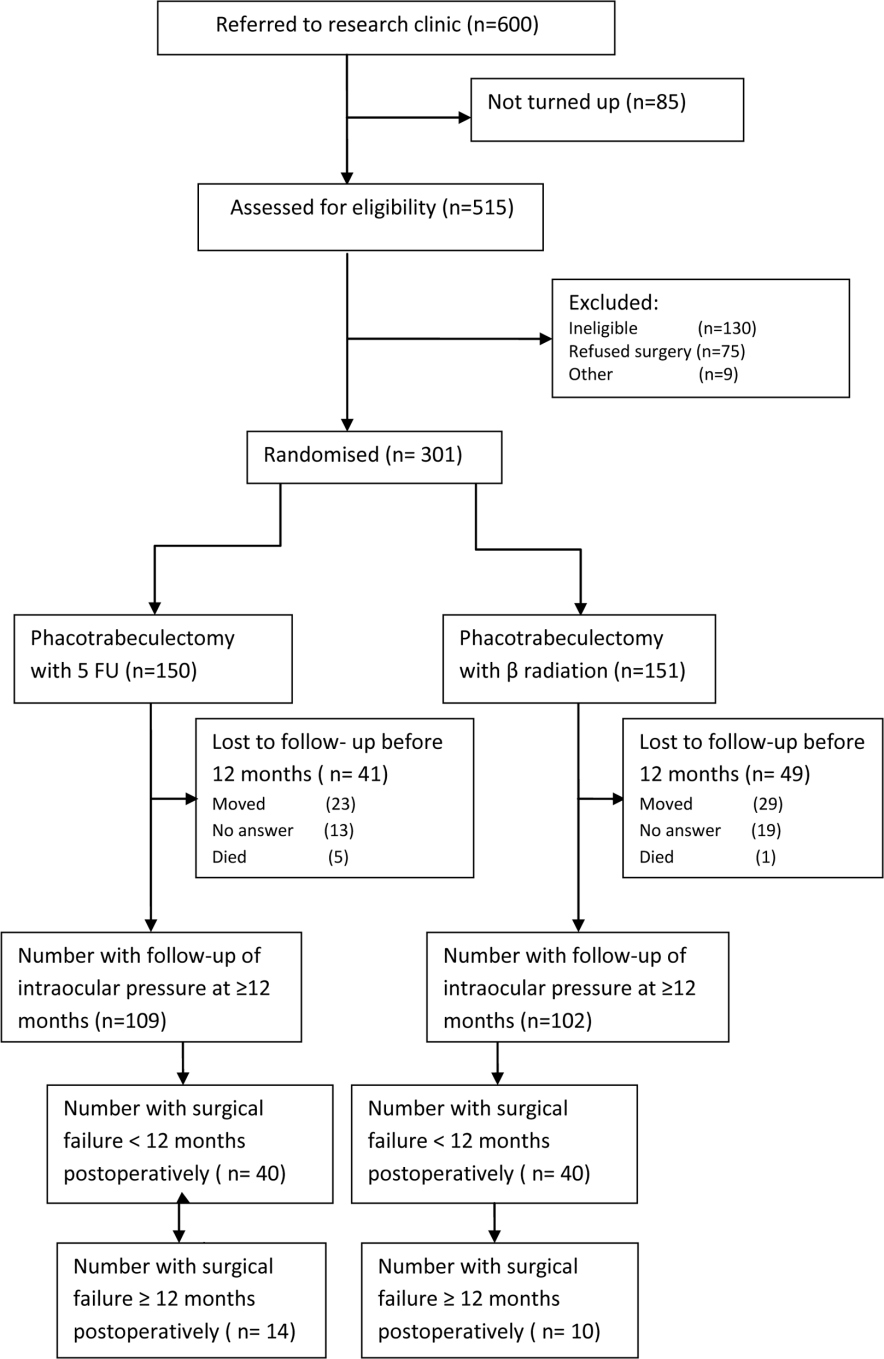
Flow diagram of recruitment and follow-up.

Overall, 301 of 385 eligible patients were randomised; 151 to the β radiation group (Fig 1). The two treatment arms were similar with respect to socio-demographic, ophthalmic and surgical factors at enrolment (Table 3). Attendance for follow-up at 10 plus months was not associated with age, gender, occupation or educational status. The most common cause for loss to follow-up was moving address.

**Table 3:** Baseline Personal, Ophthalmic and Surgical factors in the two treatment arms.

Variable	β radiation group (N = 151)	5FU group (N = 150)
	n (%)	n (%)
Age (years)		
<60	14 (9)	18 (12)
60–69	67 (44)	60 (40)
70+	70 (46)	72 (48)
Sex		
Male	104 (69)	103 (69)
Female	47 (31)	47 (31)
Education		
None	59 (39)	58 (39)
Primary	69 (46)	67 (45)
Secondary and above	23 (15)	25 (17)
Family history of glaucoma		
Yes	51 (34)	55 (37)
No	100 (66)	95 (63)
Glaucoma		
POAG	129 (85)	133 (89)
PXF	22 (15)	17 (11)
Used ocular hypotensive treatment**		
Yes	31 (21)	41 (28)
No	118 (79)	108 (73)
Mean presenting visual acuity (log Mar (95%CI))*	0.86 (0.76–0.96)	0.91 (0.80–1.01)
Mean presenting IOP (mmHg (95%CI))	34.2 (32.8–35.7)	34.9 (33.4–36.3)
Mean vertical C/D ratio (95% CI)	0.83 (0.81–0.85)	0.82 (0.80–0.84)
Mean disc height (mm) (95%CI)	2.06 (2.02–2.10)	2.05 (2.01–2.10)
Mean presenting VA (log Mar (95%CI)) fellow eye*	1.45 (1.24–1.66)	1.45 (1.25–1.64)
	

* A logMar coding for Counting Fingers = 2, Hand Motion = 3, Light Perception = 4.

** Seventy three individuals (31 beta and 42 5FU) were on IOP lowering medication immediately before surgery. The two principal drugs were topical betablockers (77%) and oral diamox (62%). 46 (79%) of the 58 on topical betablockers were taking it for less than 6 weeks preoperatively and only 4 had been on it for 1 year or more. All but 3 of the 45 on oral acetazolamide were taking it for less than two weeks pre-operatively.

### Primary outcome: surgical success

Table 4 shows the numbers of patients attending at the different follow up points throughout the study period together with the success proportion for two outcomes (IOP<16 mmHg, not on ocular hypotensive treatment; IOP≤21mmHg) and mean IOP. There was no suggestion of a difference between the two arms at any time point except 6 months, when the proportion of patients with IOPs >21mmHg was somewhat higher in the beta radiation arm (19% versus 8%; Chi^2^ = 4.79 P = 0.03) and at 12 months when the proportion of patients with IOP’s ≥16mmHg or on ocular hypotensive treatment was somewhat higher in the 5FU arm (59% vs 48%;Chi^2^ = 2.1 p = 0.15). Using GEE with an identify link function to model the probability of success across all of the observations, accounting for within individual correlation, the probability of success at any time point was estimated to be 6.7 percentage points higher in the beta arm than in the 5 Fluorouracil arm (95% c.i. -1.6% to +15.0%; P = 0.11).

**Table 4:** Surgical Success judged by two criteria IOP≤16, not on ocular hypotensive treatment and ≤21mmHg at different follow up intervals.

	Beta radiation					5 Fluorouracil					Chi^2^p-valueβ vs 5FU <16mmHg
Follow up interval	N	Success <16mmHg and not on treatment (%)	Success ≤21 mmHg (%)	Number on treatment	Mean IOP (mmHg)	N	Success<16mmHg and not on treatment (%)	Success ≤21 mmHg (%)	Number on treatment	Mean IOP (mmHg)	
1 month(15–59 days)	134	95 (70.9%)	121 (90.3%)	2	13.5	141	91 (64.5%)	120 (85.1%)	0	14.8	0.26
3 months(60–119 days)	109	75 (68.8%)	100(91.7%)	3	14.6	110	68 (61.8%)	99 (90.0%)	2	15.2	0.28
6 months(141–229 days)	97	62 (63.9%)	79 (81.4%)	1	16.1	100	58 (58.0%)	92 (92%)	3	15.6	0.40
12 months(301–429 days)	75	39 (52.0%)	68 (90.6%)	4	15.8	86	35 (40.7%)	76 (88.4%)	3	16.5	0.15
Not seen at 1 year but seen at > 1 year(>430 days)	27	11 (40.7%)	24 (88.8%)	1	17.2	23	10 (43.5%)	19 (82.6)	0	17.8	0.85

**Fig 2:**
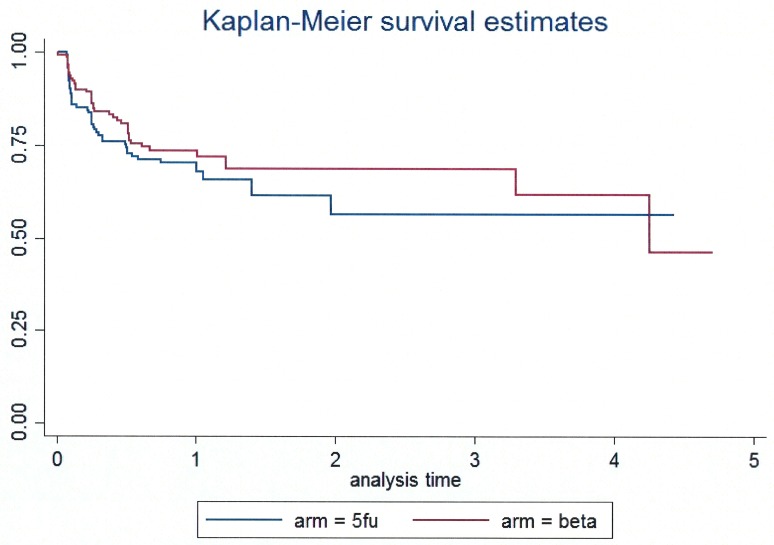
Survival analysis of IOP control in combined trabeculectomy and cataract surgery. Failure is mean IOP>21mmHg with or without medical treatment.

To facilitate comparison with a majority of literature on trabeculectomy surgery, the remaining analyses have been performed using an IOP cut-off of 21mmHg. Over the entire period of follow-up a total of 104 (34.5%) cases of surgical failure were identified (mean intraocular pressure > 21mmHg with or without ocular hypotensive treatment). The cumulative incidence of failure was similar in the two arms (log rank test p = 0.37, Fig 2). Up to day 430 there were 43 failures identified in the 5FU arm and 35 in the beta arm. Cumulative success proportions up to day 430 were 66% (95% c. i. 55% to 74%) and 72% (63% to 79%) respectively. To exactly 12 months there were 40 failures (cumulative survival 70% (62% to 77%)) in the 5FU arm and 34 failures (cumulative survival 74% (65% to 80%)) in the beta arm. The hazard ratio for the beta radiation arm compared to the 5FU arm, adjusted for IOP at baseline, was 0.83 (95% c.i. 0.54 to 1.28; P = 0.40).

If treatment success is defined as a reduction of 30% or more in IOP and an IOP≤21 mmHg, then at one year the proportion of cross-sectional successful outcomes in the 5FU and beta radiation arms were 82.6% and 82.7% respectively (P = 0.99).

### Secondary outcomes

#### Visual acuity

The mean visual acuity by arm at different time points is shown in Table 5. The beta-radiation group consistently had slightly better acuities (of the order of 0.1 logMAR from 1 month on), which could have been due to chance with the exception of Day 7, at which point there was some evidence of better visual acuity in the beta radiation group.

**Table 5:** Mean Visual acuities (log Mar) at different follow up intervals in the two treatment arms.

	Va (logMar)		t-test p value (adjusting for baseline VA)
	β	5fu	
Day 7 (3–14 days)	0.90 (95% c.i. 0.78, 1.02)	1.12 (95% c.i. 0.99, 1.25)	0.02
1 month (15–59 days)	0.74 (95% c.i. 0.63, 0.85)	0.85 (95% c.i. 0.74, 0.96)	0.27
3 months (60–119 days)	0.64 (95% c.i. 0.52, 0.76)	0.74 (95% c.i. 0.62, 0.86)	0.25
6 months (141–229 days)	0.63 (95% c.i. 0.51, 0.76)	0.73 (95% c.i. 0.62, 0.85)	0.41
12 months(301–429 days)	0.54 (95% c.i. 0.39, 0.70)	0.63 (95% c.i. 0.51)	0.53
>1 year (>430 days)	0.63 (95% c.i. 0.43, 0.82)	0.73 (95% c.i. 0.41, 1.04)	0.40

#### Complications

The per-operative complications by randomisation group are shown in S1 Table. The post-operative complications are shown in Table 6. No evidence was found of them being directly linked to subsequent surgical failure. A total of 49 (16.8%) patients had postoperative bleb needling done: 28/150 (18.7%) in the 5FU group and 21/151 (14.0%) in the β group (p = 0.26). The proportion of those who underwent needling that subsequently failed were 59% and 57% in the two treatment groups respectively.

**Table 6:** Per and Postoperative complications.

Complications	Intra operative	Post operative				
		1 week n = 300	1 month n = 275	3 months n = 219	6 months n = 197	≥ 1 year n = 211
*Major	15 (5.0%)					
**Minor	14 (4.6%)					
Bleb leak		12 (3.9%)	1 (0.4%)			
Corneal edema		115 (38%)	4 (1.5%)	2 (0.9%)	2 (1.0%)	1 (0.5%)
Shallow AC		6(1.9%)	2(0.7%)			
Hyphema		5(1.7%)				
Fibrinous AC reaction		7 (2.3%)	4(1.5%)	1 (0.5%)	1 (0.5%)	
Hypopyon		1(0.3%)				
RetainedSoft lens matter			3(1.0%)			
Vitreous hemorrhage				1 (0.5%)		
Hypotony***			3(1.0%)			
Malignant glaucoma					1 (0.5%)	
Vitritis					1 (0.5%)	
IOL capture			2 (0.7%)			

*Conjunctival button hole, vitreous loss, excessive bleeding from Iridectomy site.

**Sub-Conjunctival haemorrhage, Posterior capsular tear, Recurrent Iris prolapse, Raised intra ocular pressure.

*** No choroidal detachments were seen.

## Discussion

This is the first randomised controlled trial comparing β radiation and 5 Fluorouracil as adjuncts in combined phacotrabeculectomy surgery. It is also the first large prospective study of phacoemulsification combined with trabeculectomy in an African population.

Following the results of an earlier trial of trabeculectomy with beta radiation in South Africa [18], our sample size calculation was based on detecting superiority of beta-radiation over 5FU, which was the standard treatment at CCBRT when we initiated the trial. In a Cochrane review similar effects of 5FU and mitomycin C (MMC) [19] were reported. A subsequent review, published in 2014 suggested that mitomycin C may have a marginally bigger effect than 5 FU [20].We detected no major difference between 5 fluorouracil and beta radiation. While our trial was not powered to demonstrate equivalence of beta radiation and 5FU, our data provide no evidence that beta-radiation is less effective than 5FU, which is reassuring given the considerable supply chain and cost advantages of beta-radiation. The lack of evidence of a difference between them is consistent with the conclusions of the Cochrane reviews of trabeculectomy surgery and O’Donoghue et al who found that β radiation had a similar anti scarring effect as 5FU in their 1 year randomised trial [21]. Factors recognised to influence surgical success in trabeculectomy surgery include type of glaucoma, number and type of IOP lowering drops and conjunctival morphology. These factors were equally distributed between groups by the randomisation process.

A number of studies have reported on the outcomes of glaucoma filtration surgery in sub-Saharan African patients (S2 Table) [18,22–34]. Reported IOP success rates for glaucoma surgery at 1 year range from 46% to 91%. A consistent factor affecting outcome is duration of follow-up. Looking at outcomes between 8–24 months post-operatively, the use of adjunctive anti-scarring therapy seems to show some superiority. With no anti-scarring therapy success in the region of 70–80% seems the norm whilst the use of anti-scarring therapy generally increases this to 80–90%. Relatively little genetic variation has been reported amongst Bantu ethnic groups [35] and post-operative IOPs in a South African study of trabeculectomy with MMC for angle recession glaucoma [36] were comparable to the outcomes in an East African study (16). It therefore appears reasonable to compare the results in this study with those we found previously in South Africa (18). The South African study assessed cumulative failure. At one year the success rate was 95% in the beta-radiation group and 70% in the placebo group using an IOP cut-off of ≤21mmHg. On this basis, our finding of 75% success at one year suggests that the addition of phacoemulsification to the procedure may reduce the success of trabeculectomy surgery to levels more aligned with trabeculectomy surgery without any anti-scaring therapy. An interesting finding to note is that a majority of the time points in our study recorded success in the region of 90% in each group. By 1 year of follow-up the cumulative risk of “ever” failure estimated in the survival analysis substantially exceeds the “snapshot” risk of failure at any given time point, highlighting the care that needs to be taken when comparing cumulative risks with cross-sectional measures of success/failure. The only other report we could find of phacotrabeculectomy surgery in an African context is our own case series from CCBRT which indicated a cross-sectional success of 84% in those with 6 months or more follow-up. This is similar to the cross-sectional measures of success seen in this trial. Interestingly, although small, the prospective study by Ogata-Iwao et al had 73% cumulative success (IOP≤21) at one year for phacotrabeculectomy with mitomycin C compared to 96% success in patients with trabeculectomy alone.

Siriwardena et al compared anterior chamber reaction using flare readings (measured in photons/millisecond) after trabeculectomy and phacoemulsification surgery. Initially similar flare readings normalized to pre-operative levels in trabeculectomy eyes after 4 weeks but took 6 months to return to normal in phaco-emulsified eyes [37]. A reason for increased failure in phacotrabeculectomy may well be prolongation of the post-operative inflammatory response. Our patients remained on topical steroids for three months post-operatively; it may be that this period should be prolonged.

It is evident from this study that phacotrabeculectomy improves vision in spite of advanced glaucomatous damage. Our patients on average gained 3 acuity lines by 1 year of follow up. Of note however the fact that the maximum gain was only attained 1 year post operatively. Although this could be subject to bias as a result of differential loss to follow-up, this finding is consistent with the report of Jin et al [38]. No patient experienced a long term decrease in acuity as a result of the surgery. 5FU is well recognised as causing temporary epithelial change in some patients (20) and this may explain the decrease in acuity in that group at day 7. Any subsequent difference between the groups may be a chance finding.

This study highlights several critical points in the quest for a therapeutic strategy for the glaucomas within an African context. Firstly, there is no evidence of a difference between the use of 5FU and beta radiation as an anti-metabolite in phacotrabeculectomy surgery. Secondly phacotrabeculectomy is a successful operation that improves vision as well as controlling IOP in the majority of patients. The success of trabeculectomy in lowering IOP is reduced when combined with phacoemulsification in comparison with trabeculectomy alone. This finding has to be set against the possible need for subsequent cataract surgery in the presence of a functioning trabeculectomy which represents a second trip and expense for the patient and results in 10–61% failure of the trabeculectomy at one year post-cataract surgery[10]

We believe further work is required to delineate the outcomes and risks of other surgical options however the results presented here represent a step forward in informing the debate.

## Supporting Information

S1 File Minimum data set for study in istata format. (DTA)

S2 File Protocol. (DOCX)

S3 File CONSORT checklist. (DOCX)

S4 File Permission for study. (PDF)

S1 Table Per-operative complications by randomisation group. (DOCX)

S2 Table Studies reporting outcomes of glaucoma filtration surgery in sub-Saharan African patients. (DOCX)
