# Supplementary material for: Is Beta Radiation Better than 5 Flurouracil as an Adjunct for Trabeculectomy Surgery When Combined with Cataract Surgery? A Randomised Controlled Trial
Source: PLoS One. 2016 Sep 8;11(9):e0161674. doi: 10.1371/journal.pone.0161674 (PMC5015865; doi:10.1371/journal.pone.0161674)
Supplement: S2 File — (DOCX) [file pone.0161674.s002.docx]

**Is beta-radiation better than 5 flurouracil as an adjunct for trabeculectomy surgery when combined with cataract surgery?**

**Summary**

Glaucoma is the second most common cause of blindness in the world after cataract. Many patients with glaucoma also have some cataract. In an African context, if the cataract does not cause significant disability it would not normally warrant surgical intervention. Removal of the cataract would, none-the-less offer brighter vision and saves them surgery at a later date.

Because of the high prevalence of co-pathology (cataract and glaucoma) in our population, and also the frequency of cataract formation following trabeculectomy, we are performing an increasing number of combined glaucoma and cataract surgeries (usually combined phaco-emulsification and trabeculectomy with 5FU augmentation) and have reported our short term outcomes. Performing cataract surgery at the same time as glaucoma surgery conveys the potential advantages of saving the patient an extra operation or preventing sight loss from cataract if the patient defaults from follow up. Doing cataract surgery at the same time as the glaucoma surgery, however, has disadvantages. The major cause of failure in this surgery is post-operative scaring which seals the drainage fistula

Current best practice in many African Eye Units is to use adjunctive 5 fluorouracil at the time of surgery in order to inhibit this scarring response. The evidence base for major effectiveness of this therapy is not overwhelming. Our group have completed a randomised controlled trial of beta-radiation for trabeculectomy compared to placebo and demonstrated a striking beneficial effect. This new study is to undertake a comparison of beta-radiation with 5 fluorouracil in order to inform current best practice and to investigate if improved success can be offered with this alternative anti-scarring therapy in combined cataract and drainage surgery.

**Introduction and literature review**

The term glaucoma defines a group of conditions in which there is a characteristic optic neuropathy associated with visual field loss. Raised intraocular pressure (IOP) is recognised to be a principal risk factor. The glaucomas are the second most common cause of blindness in the world.^1 2^ There are many different types of glaucoma but about two thirds of global glaucoma is due to primary open angle glaucoma (POAG). This type of glaucoma is particularly common in Africa ^3 4^ where the condition presents earlier in life, often with severe disease.^5 6^ Because of its insidious nature, many patients present with total visual loss in at least one eye already.^7 8^ We only have one method of treating POAG at present, the lowering of IOP by medicine or surgery. There is no current method of preventing the onset of POAG.

In the very small number of studies addressing this issue, drainage surgery has been demonstrated to give superior long-term therapeutic results in terms of IOP control and visual field preservation.^9 10^ POAG in the developing world is characterised by late presentation with very advanced disease, and by the implausibility of regular patient assessment.^8^ Cost, lack of reliable medical supplies and irregular patient review render medical treatment impractical. It is generally agreed that the treatment of choice in this setting is primary drainage surgery (trabeculectomy).^11^

Cataract is the most prevalent cause of blindness globally and is also age-related. It is therefore not too surprising that patients presenting with glaucoma often have cataract as co-pathology. The therapy for cataract is cataract extraction with intraocular lens implantation. In an African context, if the cataract does not cause significant disability it would not normally warrant surgical intervention. Removal of the cataract would, none-the-less offer brighter vision and saves them surgery at a later date. It is well recognized that between 20-50% of those that have trabeculecotomy surgery alone for glaucoma require subsequent cataract surgery.

Because of the high prevalence of co-pathology (cataract and glaucoma) in our population, and also the frequency of cataract formation following trabeculectomy, we are performing an increasing number of combined glaucoma and cataract surgeries (usually combined phaco-emulsification and trabeculectomy with 5FU augmentation) and have reported our short term outcomes. Performing cataract surgery at the same time as glaucoma surgery conveys the potential advantages of saving the patient an extra operation or preventing sight loss from cataract if the patient defaults from follow up. Doing cataract surgery at the same time as the glaucoma surgery, however, has disadvantages. The major cause of failure in this surgery is post-operative scaring which seals the drainage fistula

Successful trabeculectomy aims at establishing a fistula with continual flow of aqueous from the anterior chamber to the sub-conjunctival space, from where it is removed by conjunctival and episcleral blood vessels. The major cause of trabeculectomy failure is fibrosis in the sub-conjunctival space sealing the fistula.^12 13^ Most cases of filtration failure have been found to occur within in the first few months after surgery.^14-16^ Certain groups of patients have a higher risk of surgical failure, notably patients with ocular inflammation, those who have received multiple topical medications, and African and Caribbean patients.^17-19^ Surgical success rates in African studies vary enormously between studies, but generally a much lower success rate has been reported than those in white patients.^18 20-22^

Probably the most significant change in the technique of glaucoma surgery in the past 10 years has been the introduction of agents that strongly affect the way wounds heal. The process of wound healing involves a cascade of cellular and biochemical events that ultimately results in the formation of a mature scar in the wound site. For descriptive purposes, this has been divided into an inflammatory, a proliferative and a remodeling phase, although *in-vivo* these phases are not distinct. Modulation of this process can be achieved in several ways. Corticosteroids are used post-operatively in all operations. Many units in the developed world use the cytotoxic agents 5-fluorouracil (5FU) and mitomycin C (MMC). These are most frequently applied at the time of surgery but 5-fluorouracil in particular may be given post-operatively in addition. Siriwardena and co-workers have recently demonstrated the use of a recombinent monoclonal antibody against transforming growth factor-beta (TGF-β) to modulate scarring.^29^Finally, beta-irradiation has been used.

Beta radiation is a particulate radiation consisting of high-speed electrons of negligible mass and one negative charge. Because of its low mass, beta radiation is rapidly attenuated by biological tissues making it very useful for superficial radiation treatments where deep tissue penetration is undesirable.^30^ In-vitro cell proliferation studies have shown that ocular fibroblast proliferation is significantly inhibited by beta radiation at doses between 500 and 1000 cGy.^31 32^ The period of growth arrest persists in-vitro for up to 28 days after doses >500 cGy, with no late recovery in cell proliferation.^33^ In addition, these growth arresting doses of radiation do not inhibit many of the other wound healing functions of the fibroblasts such as migration, contraction and extracellular matrix deposition, unlike the effects of 5-FU and MMC.

That Beta irradiation reduces wound healing has been known for over sixty years. Its effects on ocular wound healing have been studied since the 1950’s. The most common and well-described application has been in the prevention of recurrence following excision of pterygia. The use of Beta irradiation in glaucoma drainage surgery was first described in 1944, and has been used on a limited basis since this time. Beta irradiation augmented trabeculectomy has been shown to provide good clinical results in congenital glaucoma a condition associated with a high rate of surgical failure.^34^ A study in ‘high risk’ eyes demonstrated a clinical effect similar to 5-FU but with significantly fewer avascular cystic blebs- a potential risk factor for late infection.^35^ Beta irradiation is applied at the conclusion of surgery by means of a Beta irradiation-emitting device. It offers many potential advantages in the treatment of POAG in the African continent.

1. Once initial capital costs are met the Beta irradiation probe has a life of approximately twenty years. In the African setting this has enormous advantages over the ongoing supply costs and logistics of 5FU, MMC and monoclonal antibodies.
2. Beta irradiation is easy to apply following trabeculectomy surgery. Precautions to prevent leakage of cytotoxic at the wound site are not required. The probe does not need sterilisation.
3. Beta irradiation has the longest therapeutic record of any method of modulation of ocular wound healing. At Moorfields, Beta irradiation has been used for well over twenty years in both adult and childhood glaucomas. There is no excess morbidity due to cataract and a very low incidence of bleb related infection (unlike MMC). The efficacy has, however, never been subject to scrutiny in a randomised trial with sufficient power.
4. The conjunctival drainage bleb morphology is generally superior, being diffuse and not thin, cystic and avascular like an MMC bleb and many 5FU blebs.

Because of these very important potential advantages of this form of therapy a large Wellcome Trust supported trial was undertaken in South Africa. This trial compared trabeculectomy augmented with 1000 cGy beta radiation with trabeculectomy. There is very strong evidence that beta radiation reduces substantially the risk of IOP failure following surgery ( P<0.0001). At one year post-surgery, the estimated risk of failure was 30% (95% c.i. 22-38%) in the placebo arm compared with 5% (95% c.i. 2-10%) in the beta radiation arm.

Current ‘best practice’ in Tanzania is considered the use of adjunctive 5 fluorouracil at the time of surgery. A study is therefore required to assess the relative merits of these two adjunctive therapies in glaucoma drainage surgery combined with cataract surgery.

**Methods**

***Design*** A randomised prospective double blind controlled surgical trial of trabeculectomy with either per-operative Beta irradiation or 5 fluoro-uracil augmentation combined with cataract surgery with intraocular lens implantation.

***Methods*** African patients with established primary open angle glaucoma and cataract requiring trabeculectomy and cataract extraction will be recruited in CCBRT Hospital, Dar es Salaam, Tanzania. Following randomisation, patients will undergo cataract extraction with lens implantation combined with trabeculectomy with either exposure to 1000 cGy of Beta irradiation at the conclusion of surgery with a Strontium-90 containing delivery device, or 5 minutes sub-conjunctival exposure to 5 fluorouracil by sponge application with subsequent washout prior to the surgery. Intraocular pressure will be measured at regular intervals for at least 12 months. The primary outcome measure of the study is intraocular pressure at 12 months. Secondary outcomes are visual assessment, re intervention rate, and surgical complications.

| Inclusion criteria | Exclusion criteria |
| --- | --- |
| - Consent to inclusion and participation in trial. - Characteristic glaucomatous changes in the optic disc. The presence of a focal or diffuse area of optic disc rim loss, so that the neuroretinal rim tissue in any quadrant is less than 5% of the disc diameter in that meridian. Extensive loss of neuroretinal rim tissue with marked optic disc cupping giving a cup disc ratio greater than 0.8. - A measured intraocular pressure greater than or equal to 21 mmHg on at least one visit before the time of listing for surgery as measured by Goldmann applanation tonometry. - An open angle on gonioscopy - Cataract sufficient to decrease vision and require surgical intervention. | - Unwillingness to participate in the study - Anterior segment neovascularisation - Past trauma to the eye or ocular adnexae - Retinal or optic nerve neovascularisation - Aphakia or pseudophakia - Previous ocular surgery - Uveitis - Inability/unwillingness to give informed consent - Unwillingness to accept randomisation - Patient less than 20 years of age - Pregnancy or female of childbearing age who may be pregnant at time of treatment (LMP). - Chronic use of topical or systemic steroids |

### *Patient flow* After informed consent all patients undergo a standardised examination to establish pre-operative ocular function and findings. At the time of surgery the patient is randomised (blocked (by centre) randomisation in London, contained in sealed envelopes) to receive beta-radiation or 5 fluorouracil treatment at the time of cataract surgery and standard trabeculectomy. Every effort will be made to follow all patients according to a standardised protocol at day 1, week 1 or 2, month 1, month 3, month 6 and month 12. All therapeutic failures will be followed closely to ascertain further management plans and subsequent therapeutic compliance. Follow-up will be offered beyond 12 months.

### *Outcomes* The primary outcome is surgical success at twelve months: defined as a maximum intraocular pressure less than or equal to 21 mmHg as measured using Goldmann tonometry on no ocular hypotensive therapy. Secondary outcome variables include visual function, reintervention rate, reintervention acceptance, and surgical complications.

*Sample size* Using one eye per patient, this has been calculated using the procedure described by Altman, calculating the standardised difference for binary data (surgical success rate). Assuming an estimated improvement of success from 75% to 90%. For a study with 80% power and using a significance level of 95%, two groups of 112 patients will be required. If loss to follow up of 33% is allowed for, this means that a total of 298 patients are required.

References

1. Thylefors B, Negrel AD. Global data on blindness. *Bull world Health Or* 1995;73:115-121.

2. Quigley HA. Number of people with glaucoma worldwide. *Br J Ophthalmol* 1996;80(5):389-93.

3. Buhrmann RR, Quigley HA, Barron Y, West SK, Oliva MS, Mmbaga BB. Prevalence of glaucoma in a rural East African population. *Invest Ophthalmol Vis Sci* 2000;41(1):40-8.

4. Leske MC, Connell AM, Schachat AP, Hyman L. The Barbados Eye Study. Prevalence of open angle glaucoma. *Arch Ophthalmol* 1994;112(6):821-9.

5. Cowan CJ, Worthen DM, Mason RP, Anduze AL. Glaucoma in blacks. *Arch Ophthalmol* 1988;106(6):738-9.

6. Wormald RP, Basauri E, Wright LA, Evans JR. The African Caribbean Eye Survey: risk factors for glaucoma in a sample of African Caribbean people living in London. *Eye* 1994;8(( Pt 3)):315-20.

7. Wormald R, Foster A. Clinical and pathological features of chronic glaucoma in north-east Ghana. *Eye* 1990.

8. Verrey JD, Foster A, Wormald R, Akuamoa C. Chronic glaucoma in northern Ghana--a retrospective study of 397 patients. *Eye* 1990.

9. Migdal C, Gregory W, Hitchings R. Long-term functional outcome after early surgery compared with laser and medicine in open-angle glaucoma. *Ophthalmology* 1994;101(10):1651-6.

10. Jay JL, Murray SB. Early trabeculectomy versus conventional management in primary open angle glaucoma. *Br J Ophthalmol* 1988;72(12):881-9.

11. Schwab L, Steinkuller PG. Surgical treatment of open angle glaucoma is preferable to medical management in Africa. *Soc Sci Med* 1983;17(22):1723-7.

12. Maumenee A. External filtering operations for glaucoma: The mechanism of function and failure. *Transactions of the American Ophthalmology Society* 1960:319-328.

13. Hitchings R, Grierson I. Clinico pathological correlation in eyes with failed fistulizing surgery. *Transactions of the Ophthalmology Society of the United Kingdom* 1983;103:84-88.

14. Molteno AC, Bosma NJ, Kittelson JM. Otago glaucoma surgery outcome study: long-term results of trabeculectomy--1976 to 1995. *Ophthalmology* 1999;106(9):1742-50.

15. Watson PG, Jakeman C, Ozturk M, Barnett MF, Barnett F, Khaw KT. The complications of trabeculectomy (a 20-year follow-up). *Eye* 1990;4(( Pt 3)):425-38.

16. Rollins D, Drance S. Five year follow-up of trabeculectomy in the management of chronic open angle glaucoma. *New Orleans Academy of Ophthalmology* 1981:295-300.

17. Broadway DC, Grierson I, Hitchings RA. Racial differences in the results of glaucoma filtration surgery: are racial differences in the conjunctival cell profile important? *Br J Ophthalmol* 1994;78(6):466-475.

18. Berson D, Zauberman H, Landau L, Blumenthal M. Filtering operations in Africans. *Am J Ophthalmol* 1969;67:395-398.

19. Miller R, Barber J. Trabeculectomy in black patients. *Ophthalmic Surgery* 1981;12:46-50.

20. Ben Sira I, Ticho U. Excision of tenons capsule in fistulising operations on africans. *American Journal of Ophthalomogy* 1969;68:336-340.

21. Luntz MH. Glaucoma surgery in the South African Bantu: an inter-racial study. *South African Medical Journal* 1970(Supplement):6-8.

22. Welsh NH. Failure of filtration operations in the African. *Br J Ophthalmol* 1970;54(9):594-8.

23. Araujo SV, Spaeth GL, Roth SM, Starita RJ. A ten-year follow-up on a prospective, randomized trial of postoperative corticosteroids after trabeculectomy. *Ophthalmology* 1995;102(12):1753-9.

24. Gressel MG, Parrish Rd, Folberg R. 5-fluorouracil and glaucoma filtering surgery: I. An animal model. *Ophthalmology* 1984;91(4):378-83.

25. Anonymous. Five-year follow-up of the Fluorouracil Filtering Surgery Study. The Fluorouracil Filtering Surgery Study Group. *Am J Ophthalmol* 1996;121(4):349-66.

26. Chen. Enhanced intraocular pressure controlling effectiveness of trabeculectomy by local application of mitomycin C. *Trans Asia-Pacific Acad Ophthalmol* 1983;9:172-177.

27. Katz G, Higginbotham E, Lichter P. Mitomycin C versus 5-fluorouracil in high-risk glaucoma filtering surgery. *Ophthalmology* 1995;102:1263-1269.

28. Singh J, O'Brien C, Chawla HB. Success rate and complications of intraoperative 0.2 mg/ml mitomycin C in trabeculectomy surgery. *Eye* 1995.

29. Siriwardena D, Khaw PT, King AJ, Donaldson ML, Overton BM, Migdal C, et al. A prospective randomised double masked placebo controlled trial of a novel human anti TGF-beta2 monoclonal antibody in trabeculectomy. *Ophthalmology* 2002:in press.

30. Sharma SC. Strontium-90 eye application. *Med Dosim* 1989;14(4):229-30.

31. Khaw PT, Ward S, Grierson I, Rice NS. Effect of beta radiation on proliferating human Tenon's capsule fibroblasts. *Br J Ophthalmol* 1991;75(10):580-3.

32. Nevarez JA, Parrish Rd, Heuer DK, Hajek AS, Houdek PV, Mallick KS. The effect of beta irradiation on monkey Tenon's capsule fibroblasts in tissue culture. *Curr Eye Res* 1987;6(5):719-23.

33. Constable PH. The effect of single doses of beta radiation on the wound healing functions of ocular fibroblasts [MD thesis]. University of Cambridge, 1999.

34. Miller MH, Rice NS. Trabeculectomy combined with beta irradiation for congenital glaucoma. *Br J Ophthalmol* 1991;75(10):584-90.

35. O'Donoghue H, Sanders D, Ayliffe W, Ridgway A. Strontium 90 vs 5-FU as adjunct to surgery for patients at high risk of trabeculectomy failure: A prospective randomised trial. *Invest Ophthalmol Vis Sci* 1994;35 (supp):1432 (Abstract).
