# Supplementary material for: Is Beta Radiation Better than 5 Flurouracil as an Adjunct for Trabeculectomy Surgery When Combined with Cataract Surgery? A Randomised Controlled Trial
Source: PLoS One. 2016 Sep 8;11(9):e0161674. doi: 10.1371/journal.pone.0161674 (PMC5015865; doi:10.1371/journal.pone.0161674)
Supplement: S1 Table — (DOCX) [file pone.0161674.s005.docx]

**S1 table Per-operative complications by randomisation group**

| COMPLICATIONS | β ( % ) N= 151 | 5FU (%) N= 150 |
| --- | --- | --- |
| Prolonged Phaco time | 2 (1.3) | 2 (1.3) |
| Corneal wound bur | 1 (0.7) | 0 |
| Descemet’s membrane stripped | 1 (0.7) | 0 |
| Bleeding from Trabeculectomy site | 1 (0.7) | 3 (2.0) |
| Posterior capsular tear | 1 (0.7) | 4 (2.6) |
| Conjunctival button hole | 2(1.3) | 1 (0.7) |
| Torn Intraocular lens | 4 ( 2.6) | 0 |
| Intraoperative shallowing of AC | 1 (0.7) | 1 (0.7) |
| Aphakia | 0 | 2 (1.3) |
