# Supplementary material for: Is Beta Radiation Better than 5 Flurouracil as an Adjunct for Trabeculectomy Surgery When Combined with Cataract Surgery? A Randomised Controlled Trial
Source: PLoS One. 2016 Sep 8;11(9):e0161674. doi: 10.1371/journal.pone.0161674 (PMC5015865; doi:10.1371/journal.pone.0161674)
Supplement: S2 Table — (DOCX) [file pone.0161674.s006.docx]

**S2 table:**

**Studies reporting outcomes of glaucoma filtration surgery in sub-Saharan African patients**

| Ref No. | Study | Design | N | F/Up | Results |
| --- | --- | --- | --- | --- | --- |
| 22 | - Comparing 5 FU and MMC - Nigeria, West Africa 2012 | Retrospective | - 132 eyes - 5FU 73 - MMC 59 | ≥ 1 year | - IOP<19mmHG after 1,2,3 years - 5FU: 78%, 71%, 59% - MMC: 88%, 81%, 76% - IOP <15mmHg after 1,2,3 years - 5FU: 71%, 64%, 55% - MMC: 86%, 79%, 76% - 30% Loss of VA by at least 2 Snellen lines - 42% had ECCE Post Trabeculectomy |
| 23 | - Long term comparison 5FU and MMC - Nigeria, West Africa 2008 | Retrospective | - 68 eyes - 5FU 38 - MMC 30 | >3years | - Mean final IOP: - 5FU 19.7mmHg - MMC 18.3mmHg - IOP<21 mmHG with or without medication ( Qualified success) - 5FU: 52.6% - MMC: 73.3% - IOP < 21mmHg without medication ( Complete success) - 5FU: 24.3% - MMC: 55.2% - 50% had > 2 Snellen’s line loss of acuity |
| 24 | - 5FU and releasable suture - Nigeria, West Africa 2011 | Retrospective | - 22 eyes | ≥ 72 weeks | - Mean IOP 16.9 ±5.6mmHg - Success rate 81% at the last follow up |
| 25 | - Long term results of glaucoma - Tanzania, East Africa 2000 | Community based, Cross sectional study | - 16 eyes - All had MMC | 3 years post operative | - IOP <15mmHg: 89% - Cataract in 33% |
| 26 | - Presentation and Surgical outcome of POAG - Nigeria, West Africa 2007 | Retrospective | - 71 eyes | Not specified | - 97% had IOP <21mmHG - 82% 10-15mmHg - 15% 16-20mmHg |
| 27 | - Post Trabeculectomy complication. - Nigeria, West Africa 2009 | Retrospective | - 76 eyes - 5FU 33% - None 67% | 1 year | - Mean IOP at 1 year: - 5FU 16.1 mmHg - None 18.5mmHg - No difference in IOP outcomes 5FU vs none |
| 28 | - MMC versus Placebo - Ethiopia ,East Africa 2009 | RCT | - 31 eyes MMC - 28 eyes Placebo | No mention in abstract | - No difference in post operative IOP - Higher complications in MMC group |
| 16 | - Trabeculectomy audit - Tanzania, East Africa 2005 | Retrospective | - 178 eyes | 8 months | - 5FU used in 36% - IOP ≤15mmHg : 73% - IOP≤ 21mmHg: 90% - No significant difference between 5FU and nothing - 25% lost ≥ 2 lines Snellen’s acuity |
| 29 | - Intraoperative 5- FU application in primary Trabeculectomy - Nigeria, West Africa 2003 | Retrospective | - 154 eyes | 18 months | - IOP≤ 20mmHg - 5FU: 76% - Control : 79% - IOP≤14mmHg - 5FU: 64% - Control: 39% - (p=0.018) |
| 30 | - Effectiveness of trabeculectomy - Nigeria, West Africa 2001 | Retrospective  ( 10 years) | - 433 eyes | 1 year | - 92% success |
| 31 | - Evaluation of Trabeculectomy - Nigeria, West Africa 2001 | Retrospective | - 56 eyes | No mention | - IOP<21mmHg : 74% - With medication : 96% |
| 32 | - Trabeculectomy with and without mitomycin C - Congo, Central Africa 2001 | RCT  One eye MMC  One eye none | - 22 eyes | 20 months | - Success - MMC : 81% - None: 64% - Complications; - MMC: 36.3% - None: 9% |
| 33 | - 5Fu versus placebo - Kenya, East Africa 2001 | RCT | - 68 eyes | 6 months, 2 years | - IOP At 6 months; - 5FU: 16.9mmHg - Placebo : 17.4mmHg - At 2 years, success - 5FU : 88.8 % - Placebo : 70.6% - Higher failure rate in placebo by 2.18 times - 30% Loss of acuity by 3 lines in both arms |
| 34 | - Trabeculectomy outcomes in advanced glaucoma - Nigeria, West Africa 2001 | Retrospective  No adjuncts used | - 142 eyes | Av.3 years | - IOP <22mmHg - 1 year 85% - 5 years 71% - IOP < 16mmHg - 1 year 65% - 5 years 46% - 12% loss of acuity by 2 lines |
| 18 | - β radiation versus placebo - South Africa 2006 | RCT | - 320 eyes; - β radiation - 164 (51%) - Placebo (49%) | 1 year | - Surgical failure - β : 5% - Placebo : 30% - Higher incidence of operable cataract in β group ( 16.7%) versus Placebo (2.8%) at 2 years |
