# Supplementary material for: Is Beta Radiation Better than 5 Flurouracil as an Adjunct for Trabeculectomy Surgery When Combined with Cataract Surgery? A Randomised Controlled Trial
Source: PLoS One. 2016 Sep 8;11(9):e0161674. doi: 10.1371/journal.pone.0161674 (PMC5015865; doi:10.1371/journal.pone.0161674)
Supplement: S4 File — (PDF) [file pone.0161674.s004.pdf]

P.O. Box 23310
Dar es Salaam
Tanzania

4th August, 2008

Dear Dr. Dhalla,

RE: PERMISSION TO CONDUCT A RANDOMISED CONTROLLED TRIAL AT CCBRT DISABILITY HOSPITAL

After reviewing your research proposal titled "IS BETA- RADIATION BETTER THAN 5 FLUROURACIL AS AN ADJUNCT FOR TRABECULECTOMY SURGERY WHEN COMBINED WITH CATARACT SURGERY? A RANDOMISED CONTROLLED TRIAL" and the attached ethical clearance from the National Institute for Medical Research (NIMR/HQ/R.8a/Vol.lx/717)), I am glad to inform you that the CCBRT management has granted you permission to conduct this trial at the hospital. You are expected to give us periodic feedback on the research progress as stipulated in the NIMR letter as well as a copy of the final report.

On behalf of the CCBRT management, I wish you and the entire research team all the best in your endeavors.

Sincerely,

COMPREHENSIVE COMMUNITY - BASED REHABILITATION IN TANZANIA
CCBRT
P.O.Box 23310
Dar es Salaam
TANZANIA

Brenda Msangi.

DH Director

communications@ccbrt.or.tz, www.ccbrt.or.tz, www.kuponafoundation.org, Tel: +255 (0) 22 260 1543

Patron: Hon. Anne Makinda, the Speaker of the House of Parliament
President: Dr. W. Slaa

Represented in the USA by:
KUPONA FOUNDATION
